# Comparison of proximal femoral nail antirotation (PFNA) with AO dynamic condylar screws (DCS) for the treatment for unstable peritrochanteric femoral fractures

**DOI:** 10.1007/s00590-013-1195-0

**Published:** 2013-02-27

**Authors:** Emrah Kemal Şahin, Ahmet İmerci, Hakan Kınık, Levent Karapınar, Umut Canbek, Ahmet Savran

**Affiliations:** 1 Department of Orthopaedics and Traumatology, Erzurum Palandoken State Hospital, Asagı Kosk Mah. Yakutiye, Erzurum, 25100 Turkey; 2 Department of Orthopaedics and Traumatology, Ankara University School of Medicine, Mesa Koru Sitesi, Fulya Blok No: 44, Cayyolu, Ankara, Turkey; 3 Department of Orthopaedics and Traumatology, Izmir Tepecik Education and Research Hospital, Izmir, 35000 Turkey; 4 Department of Orthopaedics and Traumatology, Mugla Sıtkı Kocman University School of Medicine, Mugla, Turkey

**Keywords:** Peritrochanteric fractures, Proximal femoral nail antirotation, Dynamic condylar screws, Intramedullary fixation, Extramedullary fixation

## Abstract

**Purpose:**

The aim of this study was to compare the results of intramedullary fixation with those of plate-screw fixation for peritrochanteric femoral fracture patients older than 60 years old.

**Methods:**

This article reports on a retrospective review of patients who had peritrochanteric femoral fractures and were treated with a 95° fixed-angle screw plate (DCS) or an intramedullary nailing system (PFNA). Patients with 79 fractures were enrolled in the study; 47 of them were treated with the PFNA system and 37 with the DCS. Followed for at least 1 year, the treatment groups were compared by taking into consideration all demographic and trauma variables.

**Results:**

No significant differences were discovered between the two groups with regard to side of injury, mechanism of trauma, associated comorbidities, AO fracture classification, average follow-up duration, mortality, and fracture reduction quality at the 1-year follow-up. The average surgical time was significantly lower in the PFNA group (57 min.) compared to the DCS group (87 min.). Longer operative time was needed in the DCS group, and thus, greater blood loss occurred compared to the PFNA group. The functional results of the PFNA group were found to be significantly better than those of the DCS group.

**Conclusions:**

Owing to some advantages, such as minimal exposure, reduced operative blood loss, and the achievement of biological fixation, PFNA is a better choice for the treatment for unstable peritrochanteric fractures.

## Introduction

Because life expectancy has increased worldwide in recent years, a considerable increase has occurred in the incidence of proximal femoral fractures [1]. These fractures usually result from minor traumas. Complications with peritrochanteric fractures arise primarily from fixation rather than union or delayed union because the peritrochanteric area is made up of spongious bones [2]. The aim of the surgery is to achieve early mobilization and to quickly return the patient to pre-surgery activity levels. However, the treatment for these fractures continues to be difficult for surgeons [3].

The best treatment for unstable peritrochanteric fractures is still a subject of debate [4]. Various implants have been designed to facilitate fracture fixation, obtain early ambulation, and reduce the risk of complications in the treatment for peritrochanteric fractures [5, 6]. These implants can be divided into two groups: intramedullary and extramedullary [5–9]. To achieve rotational and angular stability, in 2004, the proximal femoral nail antirotation (PFNA) device, one of the third-generation intramedullary implants (PFNA; Synthes^®^ Oberdorf, Switzerland), was developed by the AO/ASIF group. PFNA blades have been biomechanically proven to compact cancellous bone and achieve increased stability and thus to delay rotation and varus collapse. Biomechanical tests have also indicated significantly higher cutout resistance in osteoporotic bone compared to other widely used screw systems [6, 10–12]. The sliding hip screw has become the most widely used extramedullary implant in the treatment for hip fractures [6, 13, 14]. Some investigators, however, have reported that this implant is not proper for unstable fractures, and these investigators have supported various alternative methods of fixation for these more difficult types of fractures [14, 15]. The DCS, an implant of extramedullary fixation, which was modified from the 95° fixed-angle plate by the AO/ASIF group, is much easier to apply in that location because its screw is cannulated [16, 17].

The purpose of this retrospective study was to compare the results of the DCS and the PFNA in the treatment for unstable peritrochanteric fractures in patients older than 60.

## Patients and methods

This observational study enrolled patients who were treated in our hospital with the DCS and the PFNA for peritrochanteric fractures between January 2007 and December 2010. The inclusion criteria were radiologically diagnosed unstable peritrochanteric fractures (31-A2 and -A3 for AO/ASIF classification), age older than 60 years old, and an American Society of Anesthesiologists (ASA) score of 1–4. The exclusion criteria were pathologic fractures, poor ambulation before the trauma, polytrauma, and severe concomitant medical conditions (ASA 5). The patients underwent surgery 4–10 days (mean, 6 days) after admission. The patients were divided into two groups. Intramedullary fixation with the PFNA system (Synthes^®^ Oberdorf, Switzerland) was implemented in Group A (*n* = 42). This group was composed of 42 patients with peritrochanteric fractures (AO Classification: 31-A2 in 23 and 31-A3 in 19). Group B (*n* = 37) underwent extramedullary fixation with the DCS system (Synthes^®^ Oberdorf, Switzerland). This group consisted of 37 patients with peritrochanteric fractures (AO Classification: 31-A2 in 21 and 31-A3 in 16).

For all of the patients, background variables, including age, gender, associated comorbidities, and mechanism of trauma, were recorded. Postoperative clinical assessments were conducted using the Salvati and Wilson [18] scoring system. The number of units of blood transfused intraoperatively and postoperatively was recorded in each group (hct <27 %).

Surgery was implemented as soon as the patients’ general health conditions were suitable. Surgeons who had performed the PFNA and DCS procedures at least three times performed the operations. All of the patients were administered a preoperative intravenous injection of antibiotic cefuroxime (1 g), and general or spinal anesthesia was used in both groups. All of the fractures in Group A were treated on the operating table in a lateral decubitus position under the control of C-arm fluoroscopy, and the fractures were reduced and treated with closed reductions. The patients in Group B were treated in a supine position with open methods under the control of C-arm fluoroscopy. Antibiotic treatments continued for 2 postoperative days. The extent of anatomical reduction (<5) was classified as acceptable (5°–10° varus/valgus and/or anteversion/retroversion) or poor (>10 varus/valgus and/or anteversion/retroversion). Rehabilitation was started as early as possible after surgery, and the patients were allowed to bear as much weight as they could tolerate. All of the patients were regularly examined physically and radiographically after 6 weeks and at 3, 6, and 12 months after their operations. Radiographs of the operated hip were obtained at each follow-up visit, and the position of the implant and extent of fracture union were noted.

### Statistical analyses

Statistical analysis was performed using two tests. Student’s *t* tests were used to compare the two groups with regard to mean age, surgical time, mean follow-up duration, units of blood transfused, partial weight-bearing time, Salvati–Wilson Hip Score, and consolidation time. χ^2^ analyses were performed to compare the groups with regard to gender, side of injury, mechanism of injury, associated comorbidities, AO fracture classification, mortality at 1-year follow-up, fracture reduction quality, and complications. A difference was considered to be statistically significant when *p* < 0.05.

## Results

The etiological reasons, in order of incidence, for treatment were falls and traffic accidents, and fall frequency was not statistically significant (*p* = 0.39). The mean surgical time for patients treated with PFNA was 57 min (range 32–96 min) and was significantly lower than in those treated with DCS, in which the mean time was 87 (range 64–178) min (*p* < 0.05). The mean Salvati–Wilson hip score on final evaluation was 31 in the PFNA group, and in the DCS group, it was 26 (*p* < 0.05). In the group treated with the PFNA, the time for consolidation was significantly shorter compared to the DCS group (*p* < 0.05). In terms of associated comorbidities, no significant differences were seen between the two groups (*p* = 0.67) (Table 1).

**Table 1:** Comparison of the main characteristics of the patients included in the study and the outcomes obtained using the PFNA and the DCS devices

	PFNA (Group A; *n* = 42)	DCS (Group B; *n* = 37)	*p* values
Gender: male/female	17/25	18/19	0.46
Age (years): mean (range)	77.02 ± 7.88	72.05 ± 5.80	<0.05
Side: right/left	19/23	17/20	0.95
Mechanism of injury			
Simple fall at home	34	27	0.39
Traffic accident	8	10	
Associated comorbidities			
Hypertension	10	8	0.67
Diabetes	7	7	
Cardiovascular disease	3	4	
Neurological disease	3	3	
AO fracture classification			
A2	23	21	0.85
A3	19	16	
Surgical time (min)	57.69 ± 17.47	87.86 ± 23.71	<0.05
Mean follow-up period (months)	20.67 ± 5.32	23.19 ± 7.22	0.07
Blood transfused (units) (erythrocyte suspensions)	0.21 ± 0.42	1.78 ± 1.08	<0.05
Partial weight-bearing (days)	7.28 ± 3.97	22,27 ± 10.72	<0.05
Mortality at 1-year follow-up	4	6	0.37
Salvati–Wilson Hip Score (maximum points 40)	31.04 ± 4.64	26.11 ± 4.97	<0.05
Consolidation time (weeks)	15.71 ± 5.49	22.59 ± 10.21	<0.05

Fracture reduction was considered good or acceptable in 69 patients (37 PFNA, 32 DCS) on postoperative radiographs. There were no significant differences between the quality of reduction for both implants and fracture types (*p* = 0.83) (Table 2).

**Table 2:** Quality of fracture reduction and postoperative radiographic evaluation

	PFNA (%)	DCS (%)	*p* value
Fracture reduction quality (%)			
Good	73.9	70.2	0.83
Acceptable	14.2	16.3	
Poor	11.9	13.5	

The orthopedic and general postoperative complications are listed in Table 3. No significant differences were seen between the two groups in terms of orthopedic or general complications (*p* = 0.10 and *p* = 0.57, respectively). The mortality rate at 1 year was 9.5 % in the PFNA group, compared with 16.2 % in the DCS group. There was no statistically significant difference between the two groups in terms of the 1-year mortality rate (*p* = 0.37).

**Table 3:** Distribution of patients with complications according to the internal fixation devices

	PFNA (*n* = 42, %)	DCS (*n* = 37, %)	*p* values
Orthopedic complications			
Lateral migration of blade or screw	2	1	0.1
Cut-out	2	1	
Nonunion	0	1	
Infection	1	3	
Implant failure	0	2	
Reoperation	2	4	
General complications			
Symptomatic DVT	1	2	0.57
Decubitus	0	1	
Pneumonia	1	1	
Urinary infection	2	1	

**Fig. 1:**
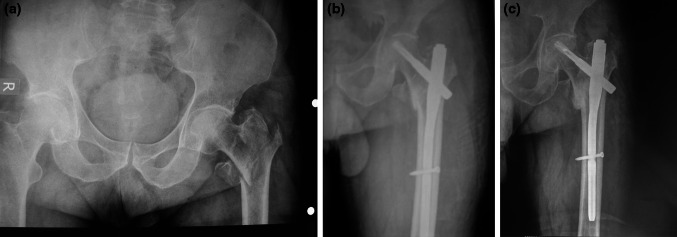
Seventy-six-year-old female patient sustaining an isolated and closed 31-A2 fracture at the *left* side (**a**) after a simple fall. Postoperative X-ray after closed reduction and internal fixation with a PFNA (**b**). Final anteroposterior hip radiograph at the 12-month follow-up (**c**)

**Fig. 2:**
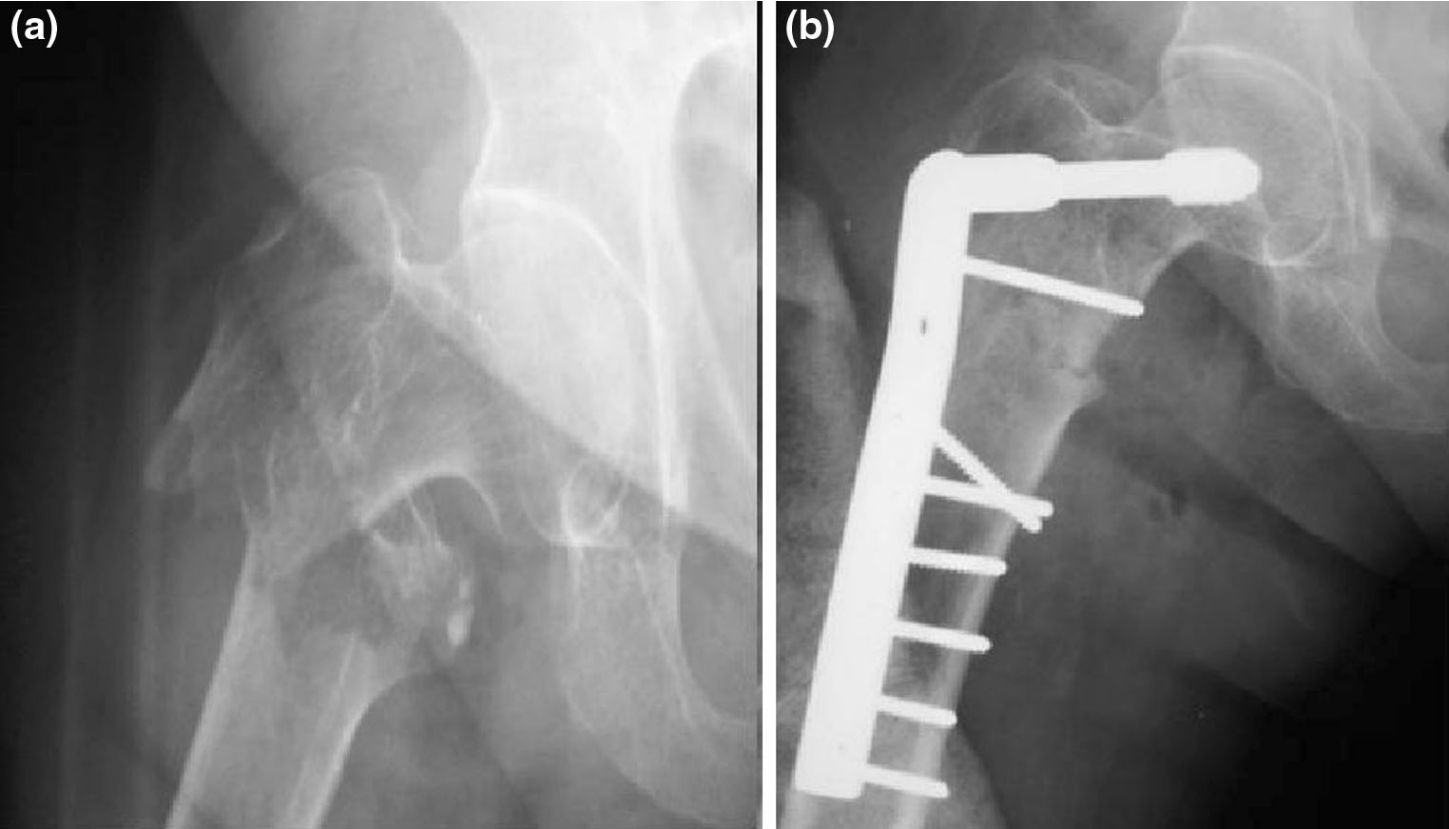
**a** Anteroposterior radiograph of the *right* hip of a 76-year-old female who fell in the street, revealing an 31-A3 peritrochanteric fracture. **b** Post op radiographs after open reduction and extramedullary fixation with a DCS

In Group A, 37 patients experienced satisfactory reduction and fixation and were able to undertake early weight-bearing. In Group B, 12 patients were able to bear weight early, although 32 patients experienced satisfactory reduction (Figs. 1, 2).

## Discussion

It is predicted that peritrochanteric femoral fractures will increase greatly in the coming years. Failure rates due to complications are still considerable, although the implants and surgical techniques have improved greatly. Rigid internal fixation combined with early mobilization is still considered the gold standard. The functional results might not be satisfactory because of failure to heal or failure of fixation, although a wide range of techniques are in use. The implants used can be partly responsible for the results. Sliding hip screws, as well as blade plates, dynamic condylar screws (DCS), and the formerly used intramedullary devices, have been found to be problematic [19]. That intramedullary devices might be superior to plating systems in unstable proximal femoral fractures has been shown in biomechanical examinations [20].

Because it produces a small bending moment, the PFNA system acts as an internal splint, and at the same time, it can bear a large axial load. Along with this ability, the helical blade of the PFNA system enhances its bone purchase in the femoral neck–head. Additionally, the blade prevents rotation or compaction of the proximal fragment by locking with the nail rotationally. These factors allow the patient to bear partial weight sooner after surgery [12, 21, 22]. Another important advantage of the PFNA technique is that it can be performed with minimal surgical invasion. Some disadvantages of this technique include cutout of the implant and femoral medialization. Lateral migration of proximal screws or helical blades is also a complication with this implant [11, 23–25].

The DCS is an implant designed by the AO/ASIF Group for use in proximal and distal femoral fractures. This device has been proved to have some technical advantages over the AO condylar blade plate [17]. DCS plates provide the ability to produce a range, especially in the sagittal plan, of rotation of the proximal part of the lag screw. However, it is obvious that many complications have been observed after surgeries. The most important of these complications are devascularization, seen as a result of over-dissection, union delay, failure to unify, and infection [26, 27]. The deficiency and fatigue of the implant should also be considered [17, 28, 29].

In our study, it was observed that the time to partial weight-bearing on the associated extremity in the DCS group was significantly longer than in the PFNA group (*p* < 0.05). In addition, the patients operated on with the DCS were more often advised to avoid sudden full weight-bearing, and because the fracture fixation was not considered stable enough, there was no satisfactory fracture impaction postoperatively [30, 31]. Open reduction and intramedullary fixation have been suggested as the first choice, regardless of age, in intertrochanteric fractures in which the medial colon is intact. IM nails, with their biomechanical features, have come into prominence in some types of unstable peritrochanteric fractures [4, 22]. In a study by Sadowski et al. [14], AO Type III patients were examined, and it was found that implant deficiency occurred in one case in the PFNA group. In our study, no significant differences between the two groups regarding orthopedic complications were detected.

The DCS used in this technique was cheaper and more widely available in our country than other techniques. There are important stages in the technique, such as appropriate placement of the guide wire and slipping of the plate over the lag screw, and these stages can be simplified using the technique explained here. Provided that the technique is performed correctly, the success rate is high [32]. As such, the technique has an important role in patients with good bone stock.

In contrast, the DCS as an extramedullary fixation device was an alternative to “intramedullary fixation” for proximal and distal femoral fracture fixations [32], although the PFNA is the gold standard for the stabilization of the femoral neck and for most peritrochanteric fractures [11, 12]. The profits of intramedullary nailing are more commonly observed than with the extramedullary procedure, which often requires reoperation due to technical problems [12].

In our series, femoral head or neck perforation was observed in three patients (3.7 %). The DCS was used in one of these patients and the PFNA in two. The rates of femoral head perforation were found to be 1.4 % in a study by Karapınar et al. and 1.2 % in a study by Simmermaher et al. [12, 23]. In a study by Sadowski et al. [14], the rates of cutout were noted as 26.3 and 5 %, respectively. In our study, the reasons for cutout in the PFNA group were related to technical failure. The blade was not in the desired central position but in the anterosuperior position. Perforation was observed in patients with the DCS because of early weight-bearing.

There are very few studies comparing intramedullary fixation with angular stable plates for the treatment for unstable fractures [14]. As in many articles in the literature, sliding hip screw devices have been compared with the PFNA in the treatment for all types of unstable intertrochanteric fractures [6, 8, 25]. In our study, there were limitations inherent in the methodology used because it was a retrospective, controlled study.

## Conclusion

The main objective of the management of elderly patients with peritrochanteric fractures is a successful return to safe mobility. In our study, the radiographic parameters were the same between the two groups. Nevertheless, intraoperative parameters, such as simpler technique, minimal exposure, shorter surgical time, reduced blood loss, and postoperative functional parameters, demonstrated that the PFNA is a more effective device for the management of peritrochanteric fractures, compared to the DCS.
